# Mesoporous activated carbon yielded from pre-leached cassava peels

**DOI:** 10.1186/s40643-021-00407-0

**Published:** 2021-06-24

**Authors:** R. Kayiwa, H. Kasedde, M. Lubwama, J. B. Kirabira

**Affiliations:** Department of Mechanical Engineering, College of Engineering, Design, Art and Technology, Makerere University, P.O. Box 7062, Kampala, Uganda

**Keywords:** Activated carbon, Cassava peel, Pre-leaching, FTIR, Surface area and pore volume

## Abstract

The search for alternatives to fossil-based commercial activated carbon (AC) continues to reveal new eco-friendly potential precursors, among which is agricultural waste. The key research aspect in all these endeavors is empirical ascertainment of the core properties of the resultant AC to suit a particular purpose. These properties include: yield, surface area, pore volume, and the active surface groups. It is therefore pertinent to have process conditions controlled and tailored towards these properties for the required resultant AC. Pre-leaching cassava peels with NaOH followed by KOH activation and carbonization at holding temperatures (780 °C) above the melting point of K (760 °C) yielded mesoporous activated carbon with the highest surface area ever reported for cassava peel-based AC. The carbonization temperatures were between 480 and 780 °C in an activation–carbonization stepwise process using KOH as the activator at a KOH:peel ratio of 5:2 (mass basis). A 42% maximum yield of AC was realized along with a total pore volume of 0.756 cm^3^g^−1^ and BET surface area of 1684 m^2^g^−1^. The AC was dominantly microporous for carbonization temperatures below 780 °C, but a remarkable increase in mesopore volume (0.471 cm^3^g^−1^) relative to the micropore volume (0.281 cm^3^g^−1^) was observed at 780 °C. The Fourier transform infrared (FTIR) spectroscopy for the pre-treated cassava peels showed distortion in the C–H bonding depicting possible elaboration of more lignin from cellulose disruption by NaOH. A carboxylate stretch was also observed owing to the reaction of Na^+^ ions with the carboxyl group in the raw peels. FTIR showed possible absorption bands for the AC between 1425 and 1712 cm^−1^ wave numbers. Besides the botanical qualities of the cassava peel genotype used, pre-leaching the peels and also increasing holding activation temperature above the boiling point of potassium enabled the modified process of producing highly porous AC from cassava peel. The scanning electron microscope (SEM) and transmission electron microscope (TEM) imaging showed well-developed hexagonal pores in the resultant AC and intercalated K profile in the carbon matrices, respectively.

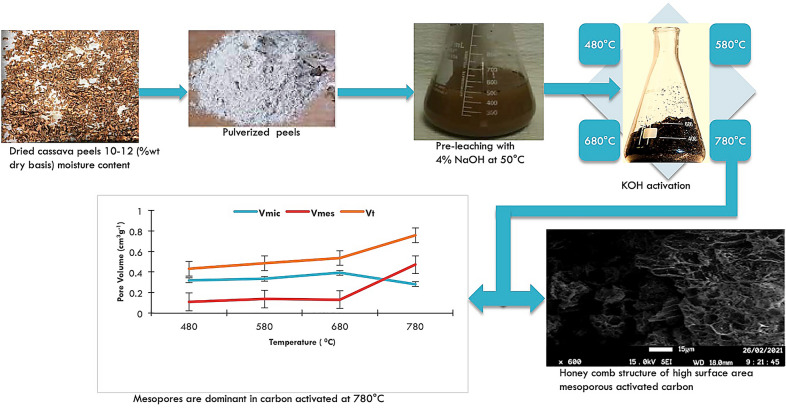

## Introduction

Activated carbon (AC) continues to find application in both domestic and industrial processes predominantly in adsorption. However, the activated carbons available on market are very costly. Besides the off-shelf cost, the non-renewable raw materials from which they are produced threaten the environment hence limiting their applications. With the global need to mitigate climate change and its eco-destructive effects, there is a need to produce AC from biomass rather than fossil sources (Yahya et al. 2015). Appreciably, research into alternative eco-friendly AC precursors has been immensely undertaken globally. Mainly, the clear-cut determinants for AC precursor choices are; availability, ease of handling, ease and cost of production, by-products' compositions, and intended application of the AC (Bhatnagar et al. 2015). In recent years, there has been a growing interest to produce ACs from lignocellulosic agricultural residues (Yahya et al. 2015). This is majorly attributed to their availability at a low cost, their high carbon content, and low inorganics. Inextricably, besides the general considerations for an AC precursor, the choice of a particular lignocellulosic residue for AC production depends on its chemical and physical properties (Menya et al. 2018). These include; moisture content, volatile matter, carbon composition, ash content, and dry matter content. Inherently, the resultant quality of the produced AC from a lignocellulosic precursor depends on the reactions and interactions of the different components of the precursor, mainly cellulose, hemicellulose, and lignin (Gani and Naruse 2007).

Cassava peel is one of the prospective precursors for AC production. Leveraging the properties of cassava peels for AC is of economic importance given the few competitive applications like animal feed (Adekunle et al. 2016; Santos et al. 2015) and, potential energy production (Okudoh et al. 2014). Like other lignocellulosic biomass, for cassava peel to be harnessed for AC production, it requires pre-treatment to counteract its recalcitrance nature towards thermochemical processing. Fairly, research into harnessing cassava peel for AC production has been conducted (Parvathi et al. 2018; Salahudeen et al. 2014; Sudaryanto et al. 2006). Of note, is the microporous structure of all the resultant ACs from these studies besides the surface area at < 1600m^2^g^−1^. However, like with other lignocellulosic precursors, there is a need to enhance the core properties of activated carbons while not compromising the production process efficiency and environmental conservation standards. Moreover, the pore size distribution of the resulting activated carbon depends on the precursor, degree of initial impregnation of the catalyst used (Daud and Ali 2004; Kwiatkowski and Broniek 2017; Laine and Yunes 1992; Li et al. 2008; Liu et al. 2015).

More often in the application of ACs, the pore volume and surface area are considered the key characteristics that define the suitability of activated carbon. In practice, some processes involving AC utilization require higher volumes of mesopores than micropores in a given AC. Such processes include but are not limited to: adsorption of large adsorbate molecules from solutions (Alves et al. 2018), biomedical engineering, and electrochemical applications (Ibeh et al. 2019; Lu et al. 2017). Moreover, mesopores are needed for access of the micropores in processes that primarily require microporous carbons (Rodríguez-Reinoso 2006). Inextricably the active surface groups of the carbon are crucial in its application properties. Therefore, more research endeavors have been put into the surface chemistry of ACs. Hence, any attempts to enhance the porosity and surface area of a prospective AC from a precursor inextricably affect the surface groups too. In our past work, peels of six predominant cassava varieties in Uganda were found viable for AC production with varietal preferences accruing to the end use of the prospective AC (Kayiwa et al. 2021). NAROCAS 1 variety was the most suitable AC precursor owing to its highest and lowest lignin and ash contents, respectively. Moreover, alkaline pretreatment of the same showed an increment in the lignin content hence an implied high char yield. Besides, NAROCAS 1 has been reported as one of the cassava varieties in Uganda that are resistant to common viral cassava diseases (Mukiibi et al. 2019; Shirima et al. 2020). This gives hope for the sustainability of AC production owing to an assured yield of enough cassava. Prospectively there was a need to study the produced AC from the variety to understand further the effect pre-leaching and a higher holding carbonization temperature have on the resultant AC porosity, surface area, and active surface groups. Besides, in AC processing reactions and activation mechanisms are variable depending not only on the activation parameters, but also on the reactivity of various carbon sources.

This study aimed at producing high surface area mesoporous activated carbon through pre-leaching and carbonizing at temperatures above that of the boiling point of the activating reagent base metal.

## Materials and methods

### Preparation of activated carbon

Activated carbon was prepared from dry peels of NAROCAS 1 cassava genotype pre-leached using NaOH solution. This genotype was chosen for the study due to its robust characteristics including char yield, carbon content, bulk density, and high lignin content. The proximate and ultimate characteristics of the cassava peel genotype are reported in our earlier work (Kayiwa et al. 2021). The peels were washed with distilled water to a debris-free level by gravitational flow method. They were dried in an oven at 40 °C for 12 h to reduce the moisture content to 10–12 (%wt dry basis) and about 100 g of the dry peels was pulverized to 0.25 mm average particle size. 20 g of the pulverized peels was soaked in 150 ml of 4.0%w/v NaOH. This was followed by mixing and heating at 400 rpm and 50 °C, respectively, in a centrifuge shaker-HERMLE Z326K, Germany for 3 h, and allowing the samples to stand for 12 h. The NaOH-pretreated cassava peel sample was then transferred to a chromatographic column, with a filter at its bottom and rinsed with distilled water until a neutral pH was obtained, followed by oven drying of the sample at 105 °C for 12 h. 10 g portions of pre-leached powdered cassava peel (0.25 mm average particle size) were mixed with KOH at KOH:peel ratios of 5:2 (mass basis) and heated at 60 °C for 2 h. The resultant slurry was dried at 100 °C for 24 h and then carbonized at temperatures of 480 °C, 580 °C, 680 °C and 780 °C in triplicate in a furnace of a thermogravimetric analyzer (TA instruments Q500, UK) under nitrogen flow of 150 cm^3^/min at a ramping rate of 10 °C per minute from room temperature of about 28 °C. It was held at the respective carbonization temperatures for 2 h after which the resultant ACs were washed with hydrochloric acid. These were sequentially put in chromatographic columns and washed with warm distilled water to a pH of 6.9. The samples were then dried in an oven at 100 °C for 12 h and kept in separate aluminum bottles for the subsequent analysis.

### Activated carbon characterization

#### Yield and bulk density

The resultant ACs were packed into a pre-weighed cylindrical crucible of mass *m*_1_ and trimmed to the top to flash with the top most rim of the crucible. The filled crucible was weighed and the mass recorded as *m*_2._ The sample mass, *m*_3_ was obtained from; *m*_3_ = *m*_2_ − *m*_1_*.* This was repeated thrice for every AC produced at a given carbonization temperature.

The inside diameter (*d*) and the height (*h*) of the crucible were measured using a vernier caliper and the volume calculated as per Menya et al. (2018):

1 $$v = \pi \left( {d/2} \right)^{{2~}} h,$$

2 $${\text{Bulk density}},\left( {{\text{kg/m}}^3} \right){\text{ was calculated from}} = \frac{{m_{3} }}{v} \times 1000.$$

The yield of the resulted activated carbon was expressed in percentage and calculated based on Eq. (3) by Omotosho and Amori (2016) as:

3 $$\frac{{{\text{Weight of activated carbon}}}}{{{\text{Weight of raw peels}}}} \times 100\% .$$

#### Specific surface area, pore size, distribution and volume

The specific surface area and the pore volumes were determined from nitrogen isotherm at 77.3 K using the BET method and the micrometrics density functional theory (DFT) software for analysis. The BET surface area was calculated using the standard BET equation applied at a relative pressure range of 0.05 to 0.3. Argon adsorption at − 186 °C was used to study the pore distribution from the adsorption isotherms. The adsorption data were analyzed using the DFT software, which enabled independent pore size determination.

#### Functional surface group determination

Fourier transform infrared spectroscopy, FTIR, was used to study the surface functional groups of raw cassava peels, pre-leached peels and the AC produced at 780 °C so that the chemical structure of the prepared AC could be determined. IR spectra were obtained with FTIR spectrometer (Shimadzu, model FTIR-8300, Japan) using the transformation of 20 scans with a spectral resolution of 4 cm^−1^ by attenuated total reflectance method. FTIR spectra were collected in the mid-infrared region between 4500 and 500 cm^−1^. Spectra were acquired using air background correction.

#### AC morphology

The morphologies of the raw peels, pre-leached peels and the resultant powdered AC carbonized at 480, 580, 680, and 780 °C were coated with gold using the sputtering technique and observed at a beam energy of 15 kV on a FEI Quanta 600, USA, scanning electron microscope (SEM). For AC produced at 780 °C, the formed briquette was analyzed both on the outer surface and on the cross-sectional cut-away surface using the same SEM and a JEOL 2100F, USA, transmission electron microscope (TEM). The surface of the AC briquette was analyzed before and after washing with water. The milled AC from peels with and without pre-leaching was also analyzed to ascertain and compare the pore development. Analyzing the 780 °C AC in briquette form was meant to ascertain the intercalation behavior of K above its boiling point.

## Results and discussion

### Bulk density

**Fig. 1:**
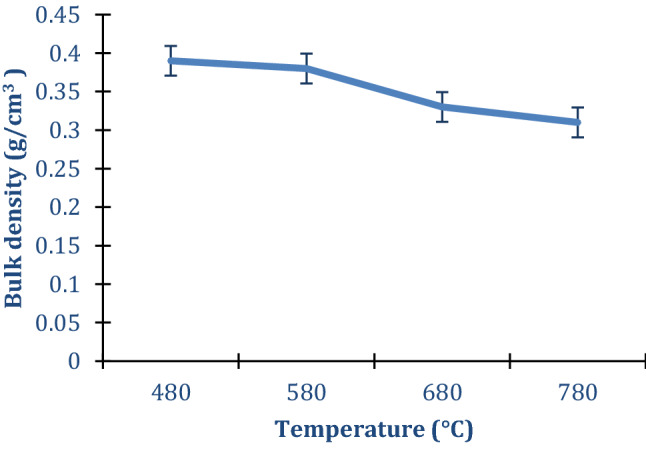
AC bulk density variation with carbonization temperature

The bulk density for the resultant activated carbon was in the range 0.31–0.39 gcm^−3^ (as shown in Fig. 1) which corroborates well with that reported by Parvathi et al. (2018), but lower than 0.410–0.415 gcm^−3^ reported by Omotosho and Sangodoyin (2013). Bulk densities of AC yielded from cassava peels without pre-leaching are in the same ranges as those of AC in this study (Table 1) at carbonization temperature ranges of 480–680 °C. At lower carbonization temperature ranges, intercalated potassium remains in the carbon matrix. This compensates for the lost inorganic content due to pre-leaching and contributes to the carbon density. Besides, at such temperatures, some interstitial spaces may still be closed hence less release of volatile matter (Molina-Sabio and Rodríguez-Reinoso 2004). Bulk density is important when estimating the packing volume and the right grade for a new system design or modification of an existing system. Powdered carbons used for decolorization usually have a bulk density in the range 0.25–0.75 gcm^−3^ while granular grades used in gas adsorption have a bulk density of around 0.40–0.50 gcm^−3^ according to the ASTM D2854-70 standard.

**Table 1:** Comparison of the bulk densities of the AC produced with those from other studies

Precursor	AC bulk density (gcm^−3^)	Intended application	References
Cassava peel	0.415	Effluent treatment	Omotosho and Sangodoyin (2013)
Cassava peel	0.31–0.39	–	This study
Cassava peel	0.36–0.48	Absorption of textile dyes	Parvathi et al. (2018)
Cassava peel	0.407–0.413	Water decontamination	Omotosho and Amori (2016)
Hard wood	0.55–0.88	–	Rodriguez-Reinoso (2002)

The highest surface area AC produced at 780 °C had the lowest bulk density that could limit its application in some processes like adsorption and gas storage. The reduction in bulk density with increasing temperature could be due to the opening of the interstitial spaces (microspores) in the carbon residue coupled with release of a great quantity of the volatile matter at high carbonization temperature.

A trade-off of high porosity and surface area for lower bulk densities has been reported for chemically activated carbons (Casco et al. 2015; Wang and Kaskel 2012). Bulk density is also important in AC intended for a number of application in capacitors. Lower bulk densities imply reduced volumetric capacitance in super capacitors despite the much required high surface area and pore volumes such ACs might have (Wang and Kaskel 2012). The lower bulk density could also have a cost implication in absorption applications since more bed volumes could be needed per unit absorbate.

### Yield of activated carbon

**Fig. 2:**
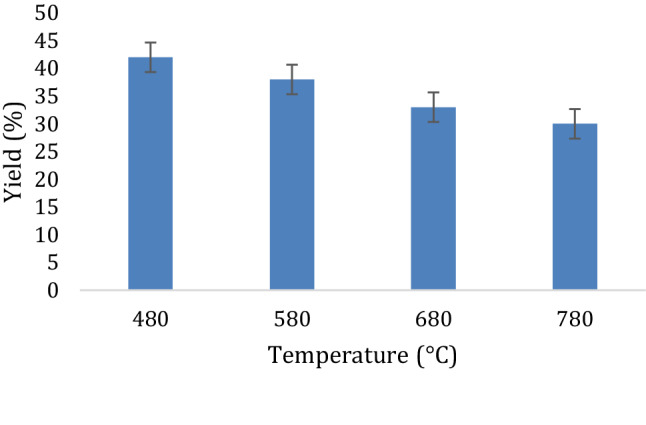
Variation of AC yield with carbonizing temperature

Results on the AC yield are presented in Fig. 2. The highest AC yield was 42% at 480 °C. This is higher than 35% the maximum value reported by Sudaryanto et al. (2006) at 1:1 impregnation ratio, carbonization temperature of 450 °C and carbonization time of 1 h without pre-leaching. The yield reduced with increase in carbonization temperature, which is in agreement with other studies (Ekebafe et al. 2012). This is attributed to the carbon burn-off and the higher reaction rate of carbon and KOH to release more volatile components with inherent improvement in the textural properties (Saka 2012). Other studies have reported reduction in AC yield due to increase in activation temperatures beyond certain limits (Kalderis et al. 2008; Sudaryanto et al. 2006; Yahya et al. 2015). For example, Sudaryanto et al. (2006) reported that the yield of the activated carbon at carbonization temperatures more than 650 °C were less than fixed carbon in the raw cassava peels. Other studies involving cassava peel AC without pre-leaching also report relatively lower yields in the range of 32.0–40.5% (Ndongo et al. 2020; Nwabanne and Igbokwe 2008; Omotosho and Amori 2016). The relatively higher yield of AC at 780 °C could be attributed to the pre-leaching of the peels in this study. The raw peel ash content is less than 5% db, the typical ash percentage composition for AC precursors (Menya et al. 2018).

However, pre-leaching reduced the ash by 0.51%, which could have contributed to an increase in char yield and subsequently upgraded to AC on activating. The small reduction in ash content yet high increment in AC yield renders the resultant AC diversely usable in a number of applications that require small amounts of ash like fuel cells and super capacitors (Xu et al. 2017) and humic acid adsorption (Daifullah et al. 2004). The high AC yield is partially attributed to the remarkably high lignin content in the NAROCAS 1 cassava variety (Kayiwa et al. 2021). High char yields have been reported from lignocellulosic precursors with relatively higher lignin compositions (Barskov et al. 2019; Sharypov et al. 2002). This is due to the high carbon content constituted in lignin (González-García 2018). The thermal treatment of hemicellulose and cellulose contained in the pre-leached cassava peel at temperatures above 250 °C contributed highly to the increase in the AC yield. This is because at such temperatures, there is an onset of elimination reactions along with crosslinking reactions leading to an increase in aromaticity.

### Pore characteristics (*pore volume, pore distribution and BET surface area*)

**Fig. 3:**
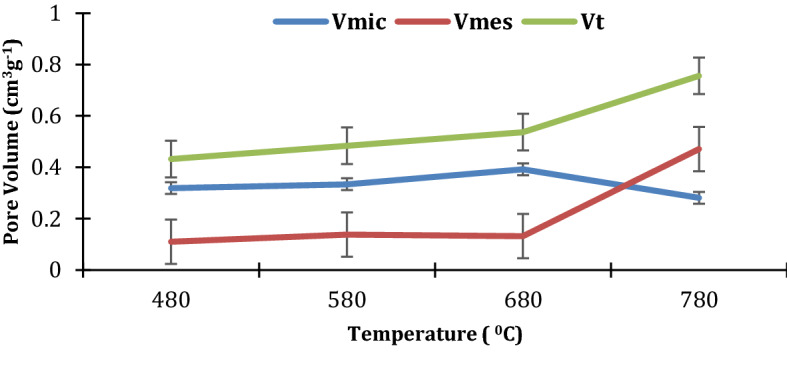
Effect of carbonization temperature on the AC pore volume

The total pore volume *V*_t_ was in the range 0.432 to 0.756 cm^3^g^−1^ and increased with increase in temperature as shown in Fig. 3. The resultant activated carbon exhibited a higher micropore volume *V*_mic_ at 0.319–0.392 cm^3^g^−1^ compared to mesopore volume *V*_mes_ for temperatures 480–680 °C. However, mesopore volume at 780 °C was remarkably higher than the micropore volume at 0.471 cm^3^g^−1^ compared to 0.281 cm^3^g^−1^. This could be due to the high lignin composition in the primary precursor. Lignocellulosic raw materials rich in lignin have been reported to yield highly mesoporous and macroporous activated carbon at temperatures higher than 650 °C (Gergova et al. 1993; Savova et al. 2001). The peels of NAROCAS 1 cassava variety were found to have relatively higher lignin compared with other predominant cassava varieties grown in Uganda (Kayiwa et al. 2021). The mesopores are important in facilitating access of the molecules to the microporosity, this being especially important in adsorption from solution processes. Other studies on deliberation of AC from cassava peels without pre-leaching reported lower pore volumes for the same or close carbonization temperature ranges. For example, Sudaryanto et al. (2006) reported total pore volumes of 0.421 to 0.519 cm^3^g^−1^ for AC carbonized at 450–650 °C. The difference could be due to the reduction in inorganic matter due to NaOH pre-leaching that enabled more spaces for volatile matter to escape during carbonization leading to more pore development. Micropores still dominate at these temperature ranges even for cassava peel AC without pre-leaching with relative increase in mesoporosity experienced with increasing temperature.

**Fig. 4:**
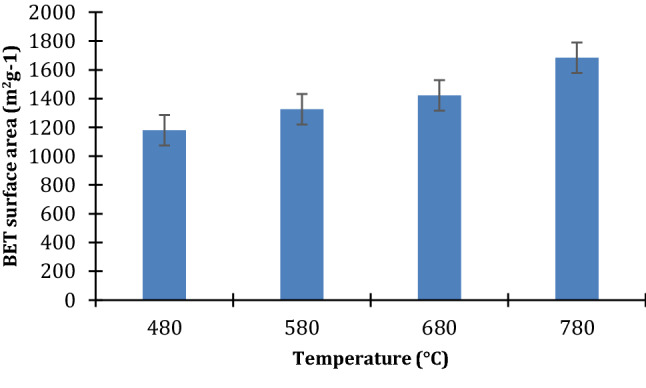
Effect of carbonizing temperature on the AC surface area

Decomposition of the lignin rich-derived char’s carbonyl, hydroxyl, and carboxyl functional groups, at high activated temperatures in the ranges 600–800 °C, produces volatile compounds (H_2_O and CO_2_) and non-volatile potassium carbonate (K_2_CO_3_) (Lozano-Castelló et al. 2007). The in situ formation of CO_2_ and H_2_O during KOH activation process plays a role in the growth of pores. Yahya et al. (2015) reported that chemical activation at higher temperatures develops the porosity of lignocellulosic samples by removing the low-molecular-weight volatile compounds from the matrix structure. The in situ physical process equations are detailed in a study by Wang and Kaskel (2012). Ramping the mixture of pre-leached Cassava peel and KOH to 780 °C and holding it at the same temperature for 2 h enabled the release of hydrogen and carbon monoxide gases. Moreover, the temperature is above the boiling point of metallic potassium (760 °C). The metallic potassium gasified and this contributed to the high porosity of the resultant AC. Besides, the intercalated metallic potassium in the carbon matrix widened the lattice spaces between carbon atomic layers (Gratuito et al. 2008; Sudaryanto et al. 2006). These in situ synergistic occurrences do not only contribute to the ordered porous structure, but also increment in surface area due to increased pore volumes. Besides, the reactivity of carbon increases with temperature and this implies more release of free K whose intercalation creates more pores notwithstanding its potential occlusion of the micropores (Huang and Zhao 2016; Liu et al. 2015). However, on washing with hydrochloric acid the blocked pores are freed enhancing the surface area as shown in Fig. 4.

**Fig. 5:**
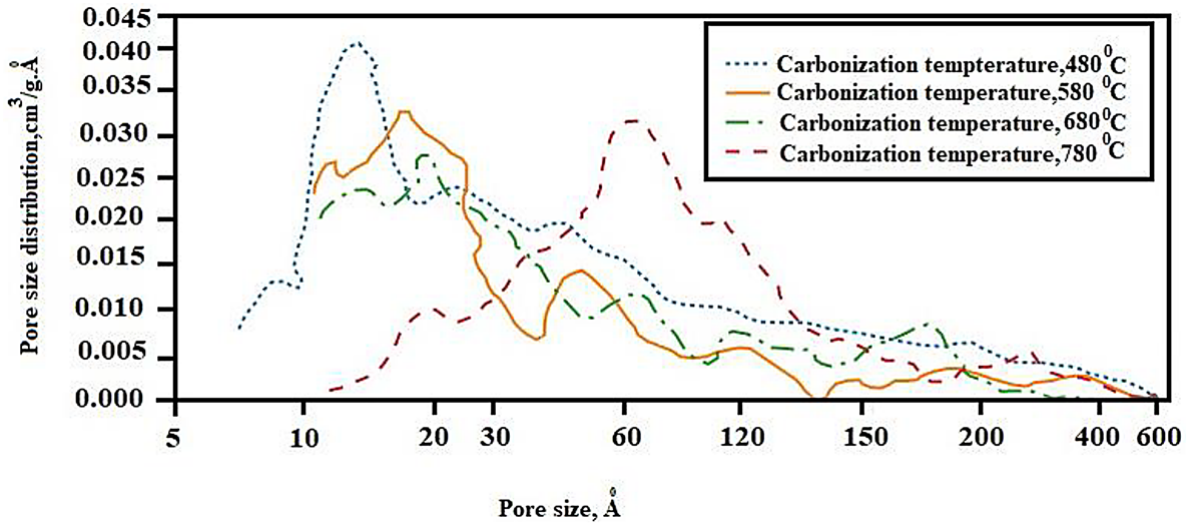
Pore size distribution for ACs produced from pre-leached cassava peels

The pore size distribution is shown in Fig. 5. At carbonization temperatures of 480–680 °C, the ACs contain micro and mesoporous structure while at 780 °C has significant mesoporous nature. This further confirms the role of the gasification of the intercalated potassium in widening the pores.

The surface area was remarkably highest at 1684 m^2^g^−1^, a value higher than 1352 m^2^g^−1^ reported by Ismanto et al. (2010) and 1567 m^2^g^−1^ by Moreno-Piraján and Giraldo (2010). This is due to the pre-leaching that reduced ash forming elements in the peels and leaving relatively higher volatile mass composition. At high carbonization temperatures the volatile content gasifies leaving voids (pores) between carbon atom layers and hence increasing the surface area. In a study by Li et al. (2008) on the effects of carbonization temperature on the porosity development in coconut shell chars and activated carbon derived from coconut shell char, the pore volumes and surface area increased appreciably with increase in temperature from 400 to 1000 °C. In the same study, surface area and pore volume for the samples at the same carbonization temperature increased with activation time (0.5 to 2 h). Moreover, KOH acts as a contraction inhibitor during carbonization. Cassava peel being lignocellulosic tends to contract more with increasing carbonization temperature. However, KOH deters such contraction, acting as a template for extra pore development (Molina-Sabio and Rodríguez-Reinoso 2004). With such a remarkable surface area, cassava peel AC could be leveraged for high-end applications like super capacitor electrodes. At 1684 m^2^g^−1^, this surface area surpasses that of 1527–1634 m^2^ g^−1^ for AC made from peanut shell and rice husk for super capacitors by He et al. (2013).

### Active surface group analysis

#### Raw peels

**Fig. 6:**
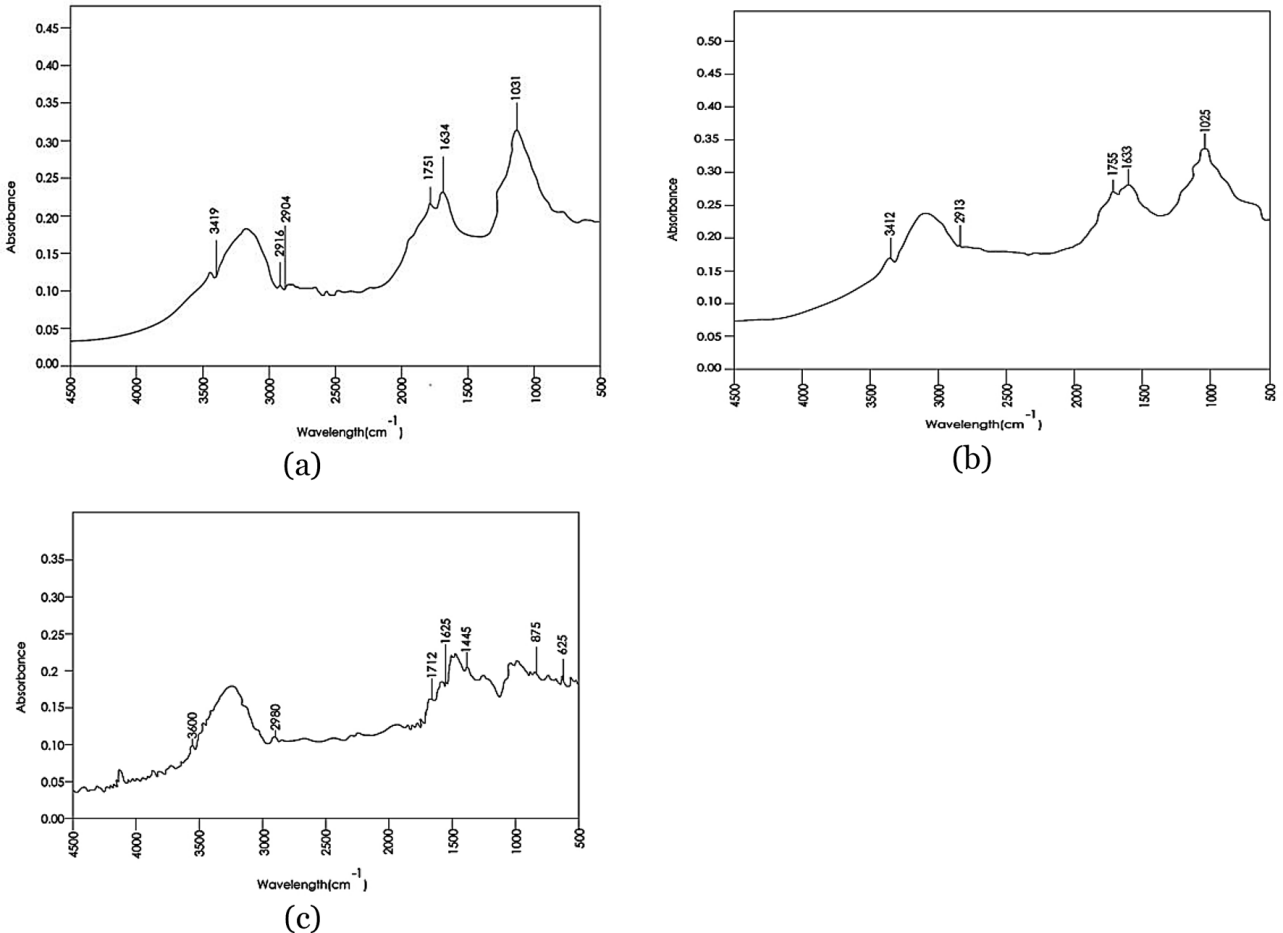
FTIR spectra for: **a** raw cassava peel, **b** pre-treated peels and **c** the resultant AC

The active surface groups have the expected main heteroatoms of carbon, hydrogen and oxygen. There are several functional groups on the raw cassava peels’ surface, namely hydroxyl, carboxyl, carboxylate and others as shown in Table 2. Similar groups were identified by other researchers (Mohd-Asharuddin, Othman, Mohd Zin, & Tajarudin, 2017; Simate, Ndlovu, & Seepe, 2015). In the same spectrum (Fig. 6a) at 1031 cm^−1^ C–O, C=C, and C–C–O stretching can be attributed to C–O–C asymmetrical stretching of hemicellulose, cellulose, and lignin.

**Table 2:** Identified surface groups from FTIR spectra compared with other studies

Material under study	Wavelength (cm^−1^)	Active surface group	References
Raw peel	3419	Intermolecular OH	This study
	2915	C–H bond from aliphatic groups	
	2904	C–H bond from aliphatic groups	
	1751	Carboxyl group stretching	
	1634	Carboxylate groups	
	1031	OH stretch (lignocellulosic precursors)	
Raw peel	500	–COOH stretch	Simate et al. (2015)
	500–1000	OH stretch	
	1500–2000	–C=O stretch	
	1000–1500	–C–O stretch	
	3000–3500	OH stretch	
Raw peel	3700–3584	O–H stretch	Idress et al. (2019)
	3550–3200	O–H stretch	
	3500	N–H stretch	
Pre-leached cassava peel	3412	O–H groups of free hydroxyl groups	This study
	2913	C–H distortion	
	2900	C–H distortion	
	1755	C–O bond of carboxyl groups	
	1633	Carboxylate group stretch	
	1025	C–O vibration	
AC from pre-leached cassava peel	3500–3600	OH group	This study
	2980	Un saturated alkynes	
	1712	Carbonyl group	
	1620	Aromatic ring	
	1445	Stretching C–C bond	
AC from cassava peel	1147	Aromatic C–H of lignin	Belcaid et al. (2020)
	1575	C=C vibration of the aromatic nucleus	
AC from cassava peel	3841.83	OH group	Astuti et al. (2020)
	1250–1170	O–C stretching	
Cassava rind carbon	1732	C=0 stretch typical of aldehydes from hemicellulose	Beakou et al. (2017)
	1156	C–O–C stretch typical of hemicellulose, cellulose, and lignin	
Cassava peel	3500–3200	O–H group in polymeric compounds such as alcohols, phenols and carboxylic acids presented in pectin, cellulose and lignin on the cassava peels	Mohd-Asharuddin et al. (2017)
	1750–1680	Stretching vibration of C=O bond of carboxyl groups	
	1300–1000	C–O stretching of COOH	
Cassava peel	3412	OH functional group	Rachman et al. (2017)
	1109	C–O vibration from secondary OH	
	3000–2750	Absorption band which is C–H sp^3^

#### Pre-leached peels

When pre-leached, the active surface groups varied according to the FTIR spectra shown in Fig. 6b. A peak at wave number 1633 of the pre-leached cassava peel indicates a carboxylate stretch most likely from the reaction of Na^+^ from NaOH with carboxyl groups in the peels. This interaction indicates potential for application as a chemisorption based adsorbent. With modifications such as Nitrogen doping, the same can be harnessed in extreme gas adsorption for example carbon dioxide capture (Liu et al. 2018). At 2913 and 2900 wave numbers, there is a distortion tendency for the C–H bond. This could be due to the disruption of hemicellulose leading to elaboration of more lignin (Sun and Cheng 2002). The distortion in the C–H bond indicates reduced crystallinity of the cellulose rich peels.

#### Resultant AC

The FTIR spectrum for the AC produced from pre-leached peels shows some band distortions as observed in Fig. 6c. Bands around 3500–3600 cm^−1^ are assigned to the stretching modes that are related to OH groups. The weak peak around 2980 cm^−1^ indicates the presence of the unsaturated alkynes C=C stretching modes. This is further coupled with a vibration of C–H bending mode around 625 to 875 cm^−1^. The band around 1620 cm^−1^ is related to the stretching vibrations of bonds of aromatic rings, those coupled with conjugated carbonyl groups on the surface (Belcaid et al. 2020). The 1712 cm^−1^ band corresponds to the stretching C=O bond whereas the band at around 1445 cm^−1^ indicates vibratory stretching of the C–C bond. The possible absorption bands for the AC were observed between 1425 and 1712 cm^−1^.

### Morphology

**Fig. 7:**
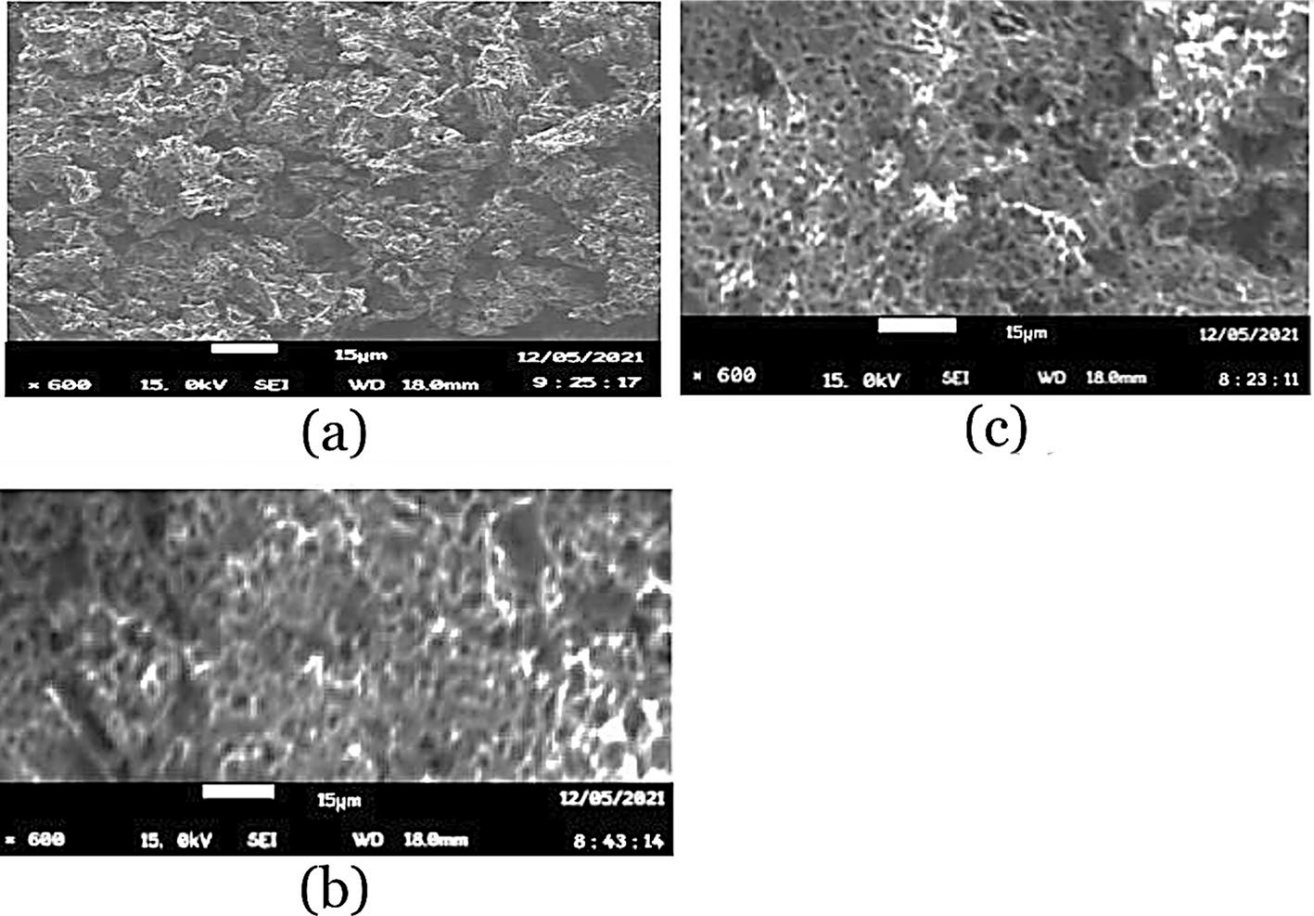
SEM micrographs for pre-leached cassava peel AC at ×600 resolution for carbonization temperatures: **a** 480 °C, **b** 580 °C, and **c** 680 °C

SEM investigated the morphology of the raw and pre-leached cassava peels and the AC produced from pre-leached peels. Raw peels exhibited heterogeneity in their structure but no porosity with starch granules numerous on the surface (Fig. 7a). The pre-leached cassava peels showed a degraded structure (Fig. 7f) compared with the raw peels. This could be due to the formation carboxylates of Na with the carboxyl group in the raw peels which distorts the starch heterogeneity through depolymerizing (Uthumporn et al. 2012). This led to a less developed porous structure composed of a few enlarged pores and a degraded surface of starch granules.

The morphology of all ACs produced at temperatures 480, 580, and 680 °C from pre-leached peels showed densely fluffy porous structures that are indicative of smaller pores as shown in Fig. 7. These structures confirm the pore distribution shown in Fig. 5 with the highest curve peaks in the microporous ranges for these temperatures. The dominance of seemingly intrinsic micropores could be due to the crystallization of K into the intra-spaces limiting pore enlargement. This is due to the inability of K to gasify below 760 °C. A slight visible variation in porosity is shown across the SEM images for the different carbonization temperatures with micropores enlarging more with increase in carbonization temperature. Increasing carbonization temperature from 480 to 680 °C effectively increases the release of volatile matter hence creating new pores and developing the existent. The structures of AC produced at or close temperature ranges from cassava peels without pre-leaching exhibit porous structures with intrinsic pores (Zdravkov et al. 2007).

**Fig. 8:**
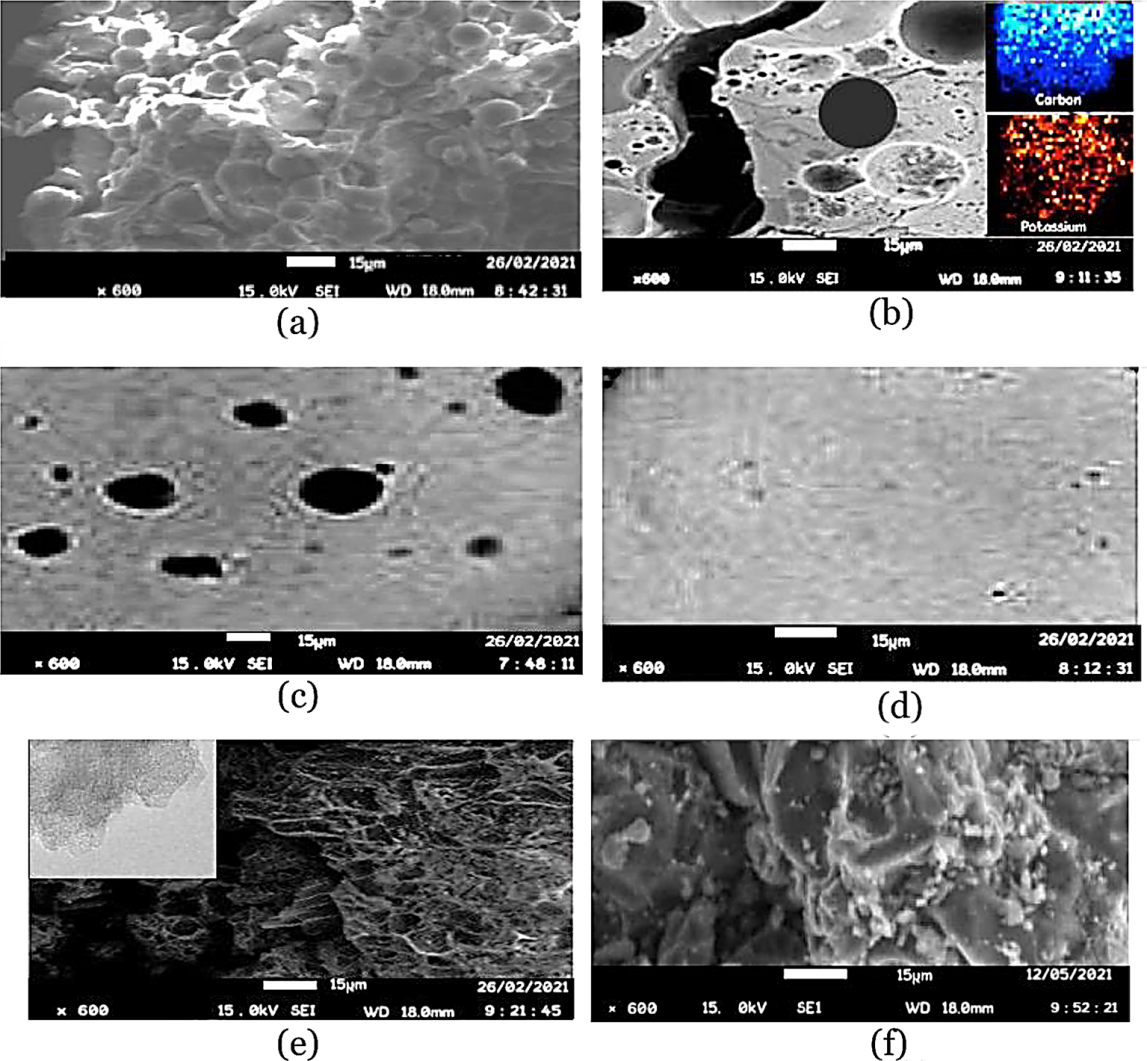
SEM micrographs at ×600 resolution and selected inset TEM images: **a** raw cassava peel; **b** cut-away surface for the washed AC briquette carbonized at 780 °C and TEM AC image (inset) for carbon and potassium, **c** outer surface of the unwashed AC briquette carbonized at 780 °C; **d** outer surface of the washed AC briquette; **e** powdered AC from pre-leached peels carbonized at 780 °C and TEM AC image (inset) for activated carbon; **f** powdered AC carbonized at 780 °C without pre-leaching

The morphology of AC carbonized at 780 °C from cassava peels without pre-leaching had a highly porous structure with intraparticle pores that appear structurally intrinsic as shown in Fig. 8f. This is due to the incorporated K into the interior of the carbon matrix, which inhibits the expected contraction with increasing temperature.

It is evident in Fig. 8 that KOH could have played a key role in pore formation by releasing free K from its reactions with carbon. The quasi-chemical bonding of carbon, oxygen and K (C–O–K) are therefore likely to have created new surface functionalities at temperatures above 700 °C hence the evidence of K species on the unwashed AC in Fig. 8c. The washed AC briquette in Fig. 8d shows that most of the free K surface species had been washed, whereas the cross-sectional view of the AC briquette in Fig. 8b as per the TEM inset showed that K species had been intercalated into the carbon matrix. This further shows that the free K that could have crystallized with in the matrix sublimed due to the temperatures above its melting point leaving free pores as seen in Fig. 8e. The species intercalated in the AC matric are hence due to the quasi-chemical bonds most likely the –O–K bond (Liu et al. 2015; Mopoung et al. 2015). The SEM image in Fig. 8e for the powdered AC showed a highly porous honeycomb structure with more extrinsic pores. This is indicative of an improved surface area (Omotosho and Amori 2016).

## Conclusions

- Pre-leaching cassava peels renders them more robust for preparation of high surface area activated carbon. This is enabled by the reduction in ash-forming agents leaving more volatile content that contributes to pore formation during carbonization. Pre-leaching also disrupts the hemicellulose hence liberating more lignin.
- Cassava peel varieties with relatively higher lignin content are more likely to produce relatively more mesoporous AC at temperatures above 650 °C not withstanding lower yields and bulk densities of the resultant AC.
- Carbonizing KOH-activated cassava peels at temperatures above the melting point of potassium improves the surface area and pore volumes. Temperatures above 760 °C enable gasification of the intercalated metallic potassium contributing to formation of more pores. However, mesopores dominate such activated carbon relative to micropores due to the already widened interstitials by the intercalated potassium. This renders the resultant activated carbon applicable in adsorption of large molecules like pharmaceuticals.

## Data Availability

The data and the materials are all available in this article as well as the supporting information.

## Abbreviations

AC: Activated carbon
FTIR: Fourier transform infrared
SEM: Scanning electron microscope
